# *cis*-1-Benzyl­pyrrolidine-2,5-dicarbonitrile

**DOI:** 10.1107/S1600536811006258

**Published:** 2011-03-02

**Authors:** Purushothama Rao Ponugoti, Narsimha Reddy Penthala, Linda P. Dwoskin, Sean Parkin, Peter A. Crooks

**Affiliations:** a Department of Pharmaceutical Sciences, College of Pharmacy, University of Kentucky, Lexington, KY 40536, USA; b Department of Chemistry, University of Kentucky, Lexington, KY 40506, USA

## Abstract

In the title compound, C_13_H_13_N_3_, the cyano groups at the 2- and 5-positions are eclipsed with each other. The phenyl ring is disordered over two sets of sites, with refined occupancies of 0.520 (5) and 0.480 (5). The angles between the mean plane of the pyrrolidine ring and the two cyano groups are 71.7 (9) and 75.0 (12)°.

## Related literature

For Robinson–Schopf condensations with succinaldehyde, see: McIntosh (1988 ▶). For lobelane (systematic name 2-[6-(2-hy­droxy-2-phenyl-eth­yl)-1-methyl-2-piperid­yl]-1-phenyl-ethanone) analog activity: Zheng *et al.* (2005 ▶).
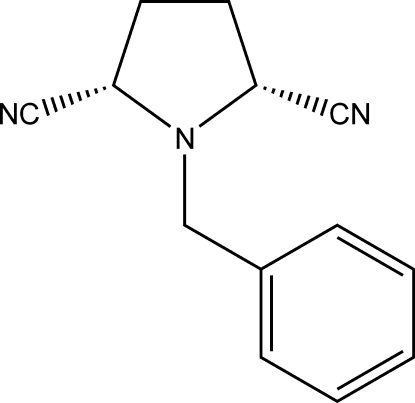


## Experimental

### 

#### Crystal data

C_13_H_13_N_3_
*M* *_r_* = 211.26
Monoclinic, 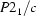

*a* = 10.9898 (3) Å
*b* = 9.4494 (2) Å
*c* = 11.4768 (3) Å
β = 103.0735 (11)°
*V* = 1160.94 (5) Å^3^
*Z* = 4
Mo *K*α radiation
μ = 0.08 mm^−1^
*T* = 150 K
0.24 × 0.20 × 0.16 mm

#### Data collection

Nonius KappaCCD diffractometer
Absorption correction: multi-scan (*SCALEPACK*; Otwinowski & Minor, 1997 ▶) *T*_min_ = 0.982, *T*_max_ = 0.988
19299 measured reflections
2662 independent reflections
1574 reflections with *I* > 2σ(*I*)
*R*_int_ = 0.061

#### Refinement

*R*[*F* ^2^ > 2σ(*F* ^2^)] = 0.051
*wR*(*F* ^2^) = 0.154
*S* = 1.04
2662 reflections
164 parameters
12 restraints
H-atom parameters constrained
Δρ_max_ = 0.17 e Å^−3^
Δρ_min_ = −0.18 e Å^−3^

### 

Data collection: *COLLECT* (Nonius, 1998 ▶); cell refinement: *SCALEPACK* (Otwinowski & Minor, 1997 ▶); data reduction: *DENZO-SMN* (Otwinowski & Minor, 1997 ▶); program(s) used to solve structure: *SHELXS97* (Sheldrick, 2008 ▶); program(s) used to refine structure: *SHELXL97* (Sheldrick, 2008 ▶); molecular graphics: *XP* in *SHELXTL* (Sheldrick, 2008 ▶); software used to prepare material for publication: *SHELXL97* and local procedures.

## Supplementary Material

Crystal structure: contains datablocks global, I. DOI: 10.1107/S1600536811006258/hg2792sup1.cif

Structure factors: contains datablocks I. DOI: 10.1107/S1600536811006258/hg2792Isup2.hkl

Additional supplementary materials:  crystallographic information; 3D view; checkCIF report

## Figures and Tables

**Fig. 1.**
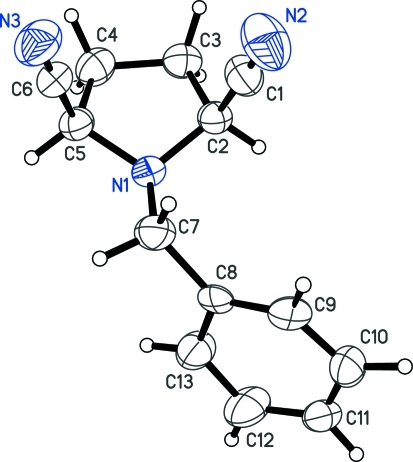
A view of the molecule with the atom numbering scheme. Displacement ellipsoids are drawn at the 50% probability level.

## supplementary crystallographic information

### Comment

With the aim of developing lobelane analogs as potent antagonists at dihydrotetrabenazine (DTBZ) binding sites on VMAT2, and as inhibiters of [^3^H]-DA uptake into vesicles (Zheng *et al.* 2005), we have undertaken the design, synthesis and structural analysis of a series of 2,5-disubstitued phenethylpyrrolidine analogs *via* a key intermediate, *cis*-*N*-benzylpyrrolidine-2,5-dicarbonitrile. The primary goal of the ananlysis of the title compound is to confirm the stereochemistry of the molecule, and to obtain detailed information on the structural conformation of the molecule. The crystal X-ray studies revealed that the central pyrrolidine ring is not planar, and that the geometry of cyano groups at the C2 and C5 positions are equatorial. The angles between the exact plane defined by N1, C2, C1 and by the mean plane passing closest to the atoms of the pyrrolidinium ring (N1, C2, C3, C4, C5), and between the exact plane defined by N1, C5, C6 to the pyrrolidinium ring (N1, C2, C3, C4, C5) for the molecule are 72.48 (9)° and 75.92 (12)° respectively.

### Experimental

The title compound was prepared by stirring a mixture of aqueous citric acid (90 ml, 0.1 *M*), 2,5- dimethoxytetrahydrofuran (8.3 mmol), benzylamine (4.5 mmol), and potassium cyanide (6.15 mmol) at ambient temperature for 48–72 h as per the literature procedure (McIntosh *et al.* 1988). The reaction mixture was then treated with excess solid sodium bicarbonate, and the reaction product extracted with dichloromethane (2x200 ml). The dichloromethane extracts were combined and dried over anhydrous sodium sulfate. The dried extract was evaporated, and chromatographed on silica gel using ethylacetate/hexane (1:9) as a eluent. From the obtained *cis* and *trans* diastereomers of *N*-benzylpyrrolidine-2,5-dicarbonitrile, the *cis*-*N*-benzylpyrrolidine-2,5-dicarbonitrile isomer was recrystallized from a mixture of diethyl ether and hexane (3:7) to afford a product which was suitable for single-crystal X-ray analysis. The 300-MHz proton spectrum of the *cis* isomer showed the benzylic protons as a clean singlet whereas in *trans* compound they appeared as an AB quartet. ^1^H NMR (CDCl_3_): δ 2.31 (*m*, 4H), 3.93 (*d*, *j*=4.1 Hz, 2H), 4.06 (*s*, 2H), 7.36 (*m*, 5H) p.p.m.; ^13^C NMR (DMSO-d_6_): δ 28.60, 51.10, 53.40, 116.90, 128.31, 128.70, 128.90, 135.20 p.p.m..

### Refinement

H atoms were found in difference Fourier maps and subsequently placed in idealized positions with constrained distances of 0.99 Å (*R*_2_CH_2_), 1.00 Å (*R*_3_CH), 0.95 Å (C_Ar_H), and with *U*_iso_(H) values set to either 1.2*U*_eq_ or 1.5*U*_eq_ (RCH_3_) of the attached atom.

The phenyl group is disordered, and *SHELXL97* commands for constraints (EADP) and restraints (SAME) were used.

### Crystal data

**Table:**

C_13_H_13_N_3_	*F*(000) = 448
*M**_r_* = 211.26	*D*_x_ = 1.209 Mg m^−^^3^
Monoclinic, *P*2_1_/*c*	Mo *K*α radiation, λ = 0.71073 Å
Hall symbol: -P 2ybc	Cell parameters from 2818 reflections
*a* = 10.9898 (3) Å	θ = 1.0–27.5°
*b* = 9.4494 (2) Å	µ = 0.08 mm^−^^1^
*c* = 11.4768 (3) Å	*T* = 150 K
β = 103.0735 (11)°	Block, colourless
*V* = 1160.94 (5) Å^3^	0.24 × 0.20 × 0.16 mm
*Z* = 4	

### Data collection

**Table:**

Nonius KappaCCD diffractometer	2662 independent reflections
Radiation source: fine-focus sealed tube	1574 reflections with *I* > 2σ(*I*)
graphite	*R*_int_ = 0.061
Detector resolution: 9.1 pixels mm^-1^	θ_max_ = 27.5°, θ_min_ = 1.9°
ω scans at fixed χ = 55°	*h* = −14→14
Absorption correction: multi-scan (*SCALEPACK*; Otwinowski & Minor, 1997)	*k* = −12→12
*T*_min_ = 0.982, *T*_max_ = 0.988	*l* = −14→14
19299 measured reflections	

### Refinement

**Table:**

Refinement on *F*^2^	Primary atom site location: structure-invariant direct methods
Least-squares matrix: full	Secondary atom site location: difference Fourier map
*R*[*F*^2^ > 2σ(*F*^2^)] = 0.051	Hydrogen site location: inferred from neighbouring sites
*wR*(*F*^2^) = 0.154	H-atom parameters constrained
*S* = 1.04	*w* = 1/[σ^2^(*F*_o_^2^) + (0.0845*P*)^2^] where *P* = (*F*_o_^2^ + 2*F*_c_^2^)/3
2662 reflections	(Δ/σ)_max_ = 0.003
164 parameters	Δρ_max_ = 0.17 e Å^−^^3^
12 restraints	Δρ_min_ = −0.18 e Å^−^^3^

### Special details

**Table:**

Geometry. All e.s.d.'s (except the e.s.d. in the dihedral angle between two l.s. planes) are estimated using the full covariance matrix. The cell e.s.d.'s are taken into account individually in the estimation of e.s.d.'s in distances, angles and torsion angles; correlations between e.s.d.'s in cell parameters are only used when they are defined by crystal symmetry. An approximate (isotropic) treatment of cell e.s.d.'s is used for estimating e.s.d.'s involving l.s. planes.
Refinement. Refinement of *F*^2^ against ALL reflections. The weighted *R*-factor *wR* and goodness of fit *S* are based on *F*^2^, conventional *R*-factors *R* are based on *F*, with *F* set to zero for negative *F*^2^. The threshold expression of *F*^2^ > 2σ(*F*^2^) is used only for calculating *R*-factors(gt) *etc*. and is not relevant to the choice of reflections for refinement. *R*-factors based on *F*^2^ are statistically about twice as large as those based on *F*, and *R*- factors based on ALL data will be even larger.

### Fractional atomic coordinates and isotropic or equivalent isotropic displacement parameters (Å^2^)

**Table:**

	*x*	*y*	*z*	*U*_iso_*/*U*_eq_	Occ. (<1)
N1	0.25240 (11)	0.12585 (13)	0.75852 (10)	0.0358 (4)	
N2	0.23554 (17)	0.03238 (18)	0.46559 (13)	0.0694 (5)	
N3	0.04139 (14)	0.30177 (17)	0.53565 (13)	0.0566 (5)	
C1	0.24055 (16)	0.01114 (17)	0.56447 (15)	0.0450 (5)	
C2	0.24688 (14)	−0.00845 (16)	0.69427 (12)	0.0359 (4)	
H2	0.3214	−0.0673	0.7306	0.043*	
C3	0.12832 (15)	−0.07866 (18)	0.71691 (14)	0.0449 (4)	
H3A	0.0811	−0.1259	0.6435	0.054*	
H3B	0.1499	−0.1499	0.7815	0.054*	
C4	0.05140 (16)	0.04144 (19)	0.75352 (14)	0.0480 (5)	
H4A	−0.0327	0.0454	0.6996	0.058*	
H4B	0.0422	0.0280	0.8366	0.058*	
C5	0.12448 (14)	0.17649 (17)	0.74314 (13)	0.0386 (4)	
H5	0.1189	0.2424	0.8099	0.046*	
C6	0.07641 (14)	0.24816 (17)	0.62628 (14)	0.0408 (4)	
C7	0.34472 (15)	0.23004 (18)	0.73940 (15)	0.0454 (4)	
H7A	0.3261	0.3209	0.7747	0.054*	
H7B	0.3324	0.2451	0.6521	0.054*	
C8	0.4794 (12)	0.1971 (13)	0.7882 (9)	0.0289 (16)	0.520 (5)
C9	0.5668 (13)	0.1649 (14)	0.7247 (11)	0.0361 (17)	0.520 (5)
H9	0.5473	0.1805	0.6408	0.043*	0.520 (5)
C10	0.6835 (16)	0.110 (3)	0.7786 (13)	0.0491 (6)	0.520 (5)
H10	0.7346	0.0671	0.7319	0.059*	0.520 (5)
C11	0.7226 (12)	0.1187 (14)	0.8995 (13)	0.049 (2)	0.520 (5)
H11	0.8052	0.0920	0.9382	0.058*	0.520 (5)
C12	0.6406 (5)	0.1667 (6)	0.9648 (5)	0.0595 (12)	0.520 (5)
H12	0.6677	0.1747	1.0492	0.071*	0.520 (5)
C13	0.5194 (6)	0.2035 (6)	0.9100 (5)	0.0512 (12)	0.520 (5)
H13	0.4635	0.2334	0.9573	0.061*	0.520 (5)
C8'	0.4716 (13)	0.1665 (15)	0.8006 (9)	0.0289 (16)	0.480 (5)
C9'	0.5535 (15)	0.1343 (16)	0.7288 (12)	0.0361 (17)	0.480 (5)
H9'	0.5246	0.1279	0.6445	0.043*	0.480 (5)
C10'	0.6791 (18)	0.111 (3)	0.7824 (14)	0.0491 (6)	0.480 (5)
H10'	0.7401	0.1175	0.7357	0.059*	0.480 (5)
C11'	0.7147 (13)	0.0806 (16)	0.9011 (14)	0.049 (2)	0.480 (5)
H11'	0.7986	0.0545	0.9357	0.058*	0.480 (5)
C12'	0.6276 (6)	0.0876 (7)	0.9703 (6)	0.0595 (12)	0.480 (5)
H12'	0.6494	0.0607	1.0522	0.071*	0.480 (5)
C13'	0.5090 (6)	0.1337 (6)	0.9200 (6)	0.0512 (12)	0.480 (5)
H13'	0.4508	0.1433	0.9694	0.061*	0.480 (5)

### Atomic displacement parameters (Å^2^)

**Table:**

	*U*^11^	*U*^22^	*U*^33^	*U*^12^	*U*^13^	*U*^23^
N1	0.0297 (7)	0.0376 (8)	0.0368 (7)	0.0013 (6)	0.0008 (5)	−0.0039 (6)
N2	0.0964 (14)	0.0703 (12)	0.0454 (9)	0.0083 (10)	0.0246 (9)	−0.0027 (8)
N3	0.0476 (9)	0.0639 (11)	0.0530 (9)	0.0107 (8)	0.0002 (7)	0.0081 (8)
C1	0.0508 (11)	0.0431 (10)	0.0412 (10)	0.0029 (8)	0.0104 (8)	−0.0039 (8)
C2	0.0351 (9)	0.0358 (9)	0.0346 (8)	0.0006 (7)	0.0033 (6)	−0.0013 (7)
C3	0.0418 (10)	0.0463 (10)	0.0455 (9)	−0.0057 (8)	0.0076 (7)	−0.0018 (8)
C4	0.0394 (10)	0.0592 (11)	0.0465 (9)	0.0003 (9)	0.0119 (8)	0.0037 (8)
C5	0.0332 (9)	0.0471 (10)	0.0337 (8)	0.0036 (7)	0.0039 (7)	−0.0040 (7)
C6	0.0315 (9)	0.0423 (10)	0.0456 (10)	0.0040 (7)	0.0026 (7)	−0.0053 (8)
C7	0.0397 (10)	0.0376 (9)	0.0545 (10)	−0.0036 (8)	0.0015 (7)	−0.0029 (8)
C8	0.0324 (16)	0.017 (5)	0.0372 (19)	−0.010 (2)	0.0068 (14)	0.0011 (19)
C9	0.038 (3)	0.030 (5)	0.0419 (12)	−0.017 (3)	0.0130 (12)	0.006 (2)
C10	0.0422 (14)	0.0482 (12)	0.0622 (13)	−0.0031 (10)	0.0231 (10)	−0.0089 (10)
C11	0.0317 (16)	0.044 (7)	0.0655 (13)	−0.003 (3)	0.0006 (11)	−0.009 (3)
C12	0.0422 (17)	0.090 (4)	0.0402 (12)	0.004 (3)	−0.0032 (11)	0.001 (3)
C13	0.0392 (15)	0.077 (4)	0.0370 (14)	0.003 (3)	0.0065 (10)	−0.011 (3)
C8'	0.0324 (16)	0.017 (5)	0.0372 (19)	−0.010 (2)	0.0068 (14)	0.0011 (19)
C9'	0.038 (3)	0.030 (5)	0.0419 (12)	−0.017 (3)	0.0130 (12)	0.006 (2)
C10'	0.0422 (14)	0.0482 (12)	0.0622 (13)	−0.0031 (10)	0.0231 (10)	−0.0089 (10)
C11'	0.0317 (16)	0.044 (7)	0.0655 (13)	−0.003 (3)	0.0006 (11)	−0.009 (3)
C12'	0.0422 (17)	0.090 (4)	0.0402 (12)	0.004 (3)	−0.0032 (11)	0.001 (3)
C13'	0.0392 (15)	0.077 (4)	0.0370 (14)	0.003 (3)	0.0065 (10)	−0.011 (3)

### Geometric parameters (Å, °)

**Table:**

N1—C5	1.457 (2)	C8—C13	1.369 (9)
N1—C2	1.4620 (19)	C9—C10	1.392 (10)
N1—C7	1.465 (2)	C9—H9	0.9500
N2—C1	1.1414 (19)	C10—C11	1.359 (10)
N3—C6	1.1427 (17)	C10—H10	0.9500
C1—C2	1.487 (2)	C11—C12	1.373 (9)
C2—C3	1.536 (2)	C11—H11	0.9500
C2—H2	1.0000	C12—C13	1.383 (7)
C3—C4	1.530 (2)	C12—H12	0.9500
C3—H3A	0.9900	C13—H13	0.9500
C3—H3B	0.9900	C8'—C13'	1.374 (9)
C4—C5	1.527 (2)	C8'—C9'	1.385 (10)
C4—H4A	0.9900	C9'—C10'	1.395 (11)
C4—H4B	0.9900	C9'—H9'	0.9500
C5—C6	1.489 (2)	C10'—C11'	1.361 (11)
C5—H5	1.0000	C10'—H10'	0.9500
C7—C8	1.493 (13)	C11'—C12'	1.377 (10)
C7—C8'	1.534 (14)	C11'—H11'	0.9500
C7—H7A	0.9900	C12'—C13'	1.372 (7)
C7—H7B	0.9900	C12'—H12'	0.9500
C8—C9	1.365 (9)	C13'—H13'	0.9500
			
C5—N1—C2	107.16 (12)	H7A—C7—H7B	107.2
C5—N1—C7	116.28 (12)	C9—C8—C13	117.0 (10)
C2—N1—C7	117.31 (12)	C9—C8—C7	127.1 (9)
N2—C1—C2	177.02 (17)	C13—C8—C7	115.8 (9)
N1—C2—C1	112.60 (13)	C8—C9—C10	122.3 (10)
N1—C2—C3	103.28 (12)	C8—C9—H9	118.9
C1—C2—C3	111.96 (12)	C10—C9—H9	118.9
N1—C2—H2	109.6	C11—C10—C9	118.5 (12)
C1—C2—H2	109.6	C11—C10—H10	120.7
C3—C2—H2	109.6	C9—C10—H10	120.7
C4—C3—C2	105.59 (13)	C10—C11—C12	118.8 (10)
C4—C3—H3A	110.6	C10—C11—H11	120.6
C2—C3—H3A	110.6	C12—C11—H11	120.6
C4—C3—H3B	110.6	C11—C12—C13	121.3 (7)
C2—C3—H3B	110.6	C11—C12—H12	119.4
H3A—C3—H3B	108.8	C13—C12—H12	119.4
C5—C4—C3	105.40 (13)	C8—C13—C12	120.4 (7)
C5—C4—H4A	110.7	C8—C13—H13	119.8
C3—C4—H4A	110.7	C12—C13—H13	119.8
C5—C4—H4B	110.7	C13'—C8'—C9'	117.1 (10)
C3—C4—H4B	110.7	C13'—C8'—C7	125.5 (10)
H4A—C4—H4B	108.8	C9'—C8'—C7	117.3 (9)
N1—C5—C6	113.23 (12)	C8'—C9'—C10'	118.9 (11)
N1—C5—C4	103.07 (13)	C8'—C9'—H9'	120.5
C6—C5—C4	111.40 (13)	C10'—C9'—H9'	120.5
N1—C5—H5	109.7	C11'—C10'—C9'	120.4 (13)
C6—C5—H5	109.7	C11'—C10'—H10'	119.8
C4—C5—H5	109.7	C9'—C10'—H10'	119.8
N3—C6—C5	178.57 (17)	C10'—C11'—C12'	119.2 (12)
N1—C7—C8	117.7 (4)	C10'—C11'—H11'	120.4
N1—C7—C8'	104.9 (5)	C12'—C11'—H11'	120.4
N1—C7—H7A	107.9	C13'—C12'—C11'	119.4 (8)
C8—C7—H7A	107.9	C13'—C12'—H12'	120.3
C8'—C7—H7A	113.7	C11'—C12'—H12'	120.3
N1—C7—H7B	107.9	C12'—C13'—C8'	122.2 (8)
C8—C7—H7B	107.9	C12'—C13'—H13'	118.9
C8'—C7—H7B	114.9	C8'—C13'—H13'	118.9
